# CP110 and CEP135 localize near the proximal and distal centrioles of cattle and human spermatozoa

**DOI:** 10.17912/micropub.biology.000951

**Published:** 2023-09-25

**Authors:** Katerina A. Turner, Drew L. Caswell, Bryce M. McGrady, Andrea Pietras-Allen, Jessica Sedlak, Cassandra Nathan, Surya Parasuraman, Aidan P. McGann, Farzeen M. Fazili, Jonathan R. Bell, Khalil N. El Smail, Sangeetha B. Pillai, Kaelan R. Parry, Kyle P. Richardson, Kelsie Ruble, Ankit Jaiswal, Tariq A. Shah, Puneet Sindhwani, Tomer Avidor-Reiss

**Affiliations:** 1 University of Toledo, Toledo, Ohio, United States

## Abstract

Centrosomes play an important role in the microtubule organization of a cell. The sperm's specialized centrosome consists of the canonical barrel-shaped proximal centriole, the funnel-shaped distal centriole, and the pericentriolar material known as striated columns (or segmented columns). Here, we examined the localization of the centriole proteins CEP135 and CP110 in cattle and human spermatozoa. In canonical centrioles, CP110 is a centriole tip protein that controls cilia formation, while CEP135 is a structural protein essential for constructing the centriole. In contrast, we found antibodies recognizing CEP135 and CP110 label near the proximal and distal centrioles at the expected location of the striated columns and capitulum in cattle and humans in an antibody and species-specific way. These findings provide a pathway to understanding the unique functions of spermatozoan centrosome.

**Figure 1. Anti-CEP135 and Anti-CP110 antibodies labeled near the proximal and distal centrioles at the expected location of the striated columns and capitulum in cattle and human:**
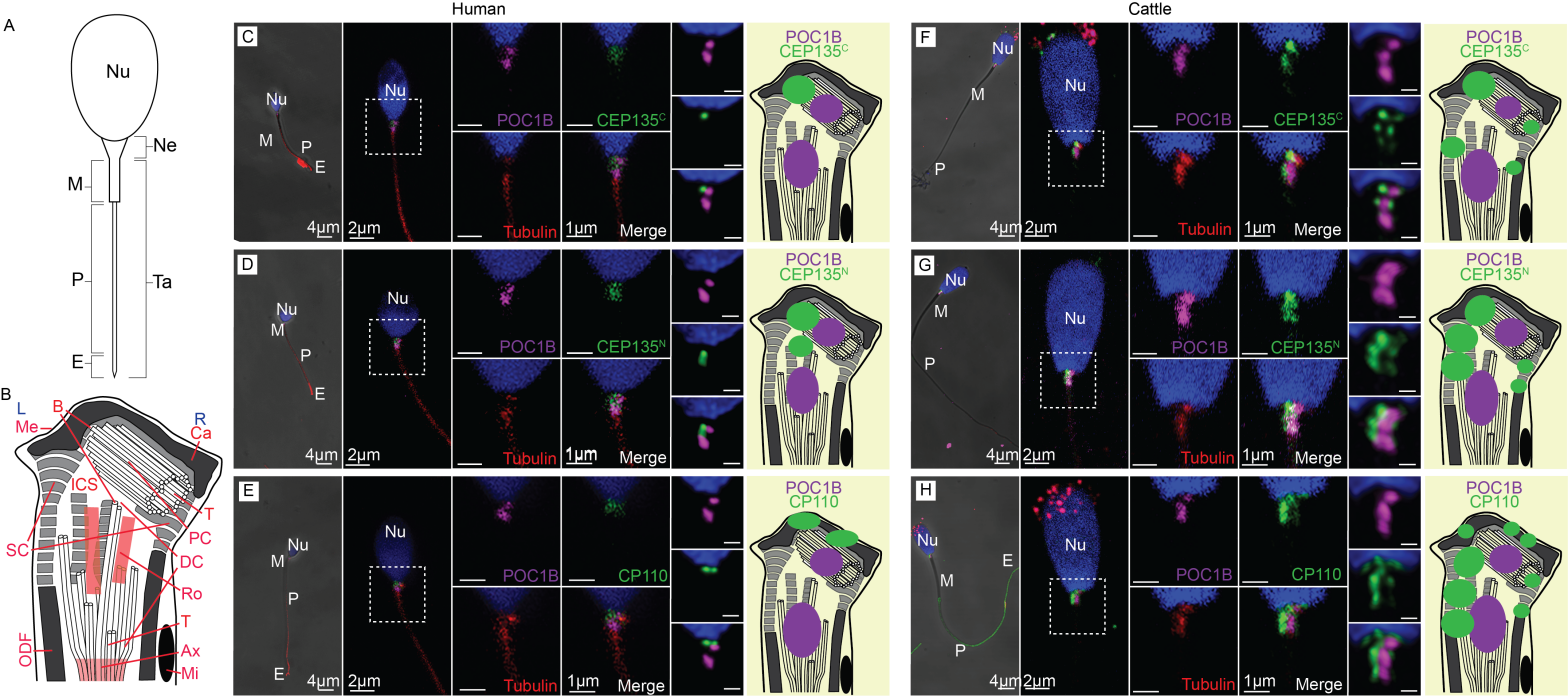
**A-B)** Drawing of a generic mammalian sperm including head with the nucleus ( **Nu** ), neck ( **Ne** ), and tail ( **Ta** ). **(A)** The tail is made of a midpiece ( **M** ), principal piece ( **P** ), and end piece ( **E** ). **(B)** The sperm neck contains the proximal centriole ( **PC** ), distal centriole microtubules ( **DC** ) and rods ( **Ro, red** ), axoneme ( **Ax** ) **,** capitulum ( **Ca** ), striated column ( **SC** ), outer dense fibers ( **ODF** ), mitochondria ( **Mi** ), and membrane ( **Me** ). Sperm left side ( **L** ) and right side ( **R** ); Centriole tip ( **T** ) and base ( **B** ); inter-centriolar space ( **ICS** ). **C-H)** Commercial (N-terminus) **(C, F)** and custom-made (C-terminus) **(D, G)** antibodies against CEP135 (green), as well as commercial antibodies against CP110 **(E, H)** (green), labeled in the vicinity of the proximal and distal centrioles in human **(C, D, E)** and cattle **(F, G, H)** spermatozoa. Tubulin (red) labels the proximal and distal centrioles in many sperms and weakly labels the axoneme. POC1B (purple) labels the proximal and distal centrioles specifically. Hoechst labels the sperm nucleus (blue). Each panel contains four groups of images from left to right: a low magnification confocal image (scale bar 4 µm), a medium magnification image in the middle (scale bar 2 µm), four high magnification images on the right (scale bar 1 µm) from the same spermatozoa, and three HyVolution images highlight the location of the experimental antibody relative to POC1B labeling from a separate spermatozoon (scale bar 0.5 µm). A drawing depicting the localization patterns of the studied proteins (green) relative to the centriolar protein POC1B (purple) associated with each panel.

## Description

The centrosome is the dominant center for microtubule organization in somatic cells and is responsible for the arrangement of microtubule arrays known as astral microtubules (reviewed in Schatten 2008). Such a centrosome consists of a pair of barrel-shaped microtubule-based structures called centrioles, surrounded by a proteinaceous scaffold known as the pericentriolar material (reviewed in Winey and O'Toole 2014). However, the two centrioles are distinct, having different sub-structures and protein compositions (reviewed in Qi and Zhou 2021). The mother centriole can form a primary cilium, which receives and transduces environmental signals to elicit cellular responses or generate cellular movement (reviewed in Joukov and De Nicolo 2019). The pericentriolar scaffold changes size throughout the cell cycle, organizing cell-cycle-specific astral microtubules.

The spermatozoan centrosome is highly specialized in structure, function, and composition and is located at the neck connecting the tail to the head ( **Figure 1A** ). It is also called the head-tail coupling apparatus (reviewed in Wu *et al.* 2020; Tapia Contreras and Hoyer-Fender 2021). It consists of two centrioles, the proximal centriole and the distal centriole, surrounded by specialized pericentriolar material, the striated column and capitulum ( **Figure 1B** ) (Reviewed in Avidor-Reiss and Fishman 2019). Most mammalian spermatozoa have a flat or pear-shaped nucleus; in a flat side view, the sperm neck exhibits left-right asymmetry ( **Figure 1B** ). By convention, the proximal centriole location determines the right side in this view. The capitulum is attached to the head via the basal plate found at the nucleus base; the distal centriole is connected directly to the axoneme; and the striated column is attached to the axoneme principal piece via the outer dense fibers (Reviewed in Avidor-Reiss *et al.* 2020). The sperm centrosome also becomes the zygote’s centrosome post-fertilization (Simerly *et al.* 1995; Uzbekov *et al.* 2023). Considering the significant structural differences between somatic and spermatozoa centrosomes, here, we aim to identify the localization of centrosome protein in the spermatozoa.

Proteomic studies have identified almost 7000 human spermatozoa proteins, of which 450 are centrosomal (Firat-Karalar and Stearns 2014; Castillo *et al.* 2018; Greither *et al.* 2023). CEP135 and CP110 have been found to localize to the general location of the proximal centriole using florescent and confocal microscopy in human spermatozoa (Sha *et al.* 2017; Fishman *et al.* 2018). Yet, the exact localization of these proteins in human and other mammal neck spermatozoa is currently unclear. Here, we aimed to study their location using both confocal (~250 nm X-Y resolution) and HyVolution (~80 nm X-Y resolution) microscopy in human and cattle spermatozoa relative to structural proteins of the centriole spermatozoa POC1B and tubulin. We were also careful to orient the spermatozoa consistently, as the spermatozoa's neck is asymmetrical (Leung *et al.* 2021). Human and cattle spermatozoa share a similar shape, but cattle atypical distal centriole is larger (Khanal *et al.* 2021). As expected, the antibodies against tubulin and POC1B in cattle and humans labeled two foci in the sperm neck, the proximal and distal centrioles (Turner *et al.* 2022) ( **Fig. 1C -H** ). POC1B, a centriole lumen protein, specifically labeled the proximal and distal centrioles. However, the confocal and HyVolution microscopy resolution did not resolve the two POC1B bars, and the labeling appeared as spots or rings. Tubulin staining was less reliable than that of POC1B. It was weak or absent in some centrioles, a property of the sheep anti-tubulin antibody we used to image multiple proteins simultaneously with indirect immunofluorescence.

Centrosomal protein 135 (aka CEP135 or BLD10) is a microtubule-binding protein with a predicted rod structure (Jumper *et al.* 2021; Varadi *et al.* 2022), that is essential for normal centriole formation (Mottier-Pavie and Megraw 2009; Bayless *et al.* 2012). CEP135 connects the cartwheel spokes and triplet microtubules, playing a role in centriole scaffolding (Lin *et al.* 2013; Noga *et al.* 2022). The cartwheel is found in the procentrioles base (Reviewed in Guichard *et al.* 2018). Furthermore, CEP135 loss of function causes spermatozoa flagella to have multiple morphological abnormalities in humans, leading to infertility (Sha *et al.* 2017).

Here, we studied CEP135 localization using two distinct antibodies. (i) Custom rabbit polyclonal antibodies against CEP135 amino acids 650-1140 (aka CEP135 ^C^ ) were provided by the Tang K Tang lab (Lin *et al.* 2013). (ii) A commercial rabbit polyclonal antibody (24428-1-AP) raised in rabbits against CEP135’s first 233 amino acids (aka CEP135 ^N^ ).

CEP135 ^C^ antibody was studied in human spermatozoa and labeled a single focus co-localizing with the centrin labeled proximal centriole, but this study did not consider the spermatozoa orientation which can affect the visualization of the colocalization (Fishman *et al.* 2018). Here, we oriented spermatozoa images for a side view, placing the proximal centriole on the right side. We found that CEP135 had a narrow distribution labeling in the human spermatozoa neck, mainly in the proximal centriole base, compared to cattle spermatozoa neck. Most CEP135 ^C^ labeled near the base of the proximal centriole ( **Figure 1C, SFig 1, SFig 2, and STable 1** ). A weaker labeling was observed near the base of the POC1B-labeled distal centriole (PC/DC= 18±32, N=30).

Similarly, CEP135 ^N^ intensely labeled near the base of the POC1B-labeled proximal centriole and weakly near the POC1B-labeled distal centriole (PC/DC): 1.58±0.96, N=30 ( **Figure 1D, SFig 3, SFig 2, and STable 2** ). Overall, both CEP135 antibodies have a common labeling near the human spermatozoa centriole base. The minor differences in the labeling pattern of the two CEP135 antibodies can be due to differences in affinity or differences in the distinct protein domains they recognize. Because POC1B labels the proximal centriole lumen both in canonical centrioles and human spermatozoa, CEP135 may label the centriole base or a nearby area.

CEP135 had a wider spread labeling in the cattle spermatozoa neck. As observed in humans, cattle CEP135 ^C^ also intensely labeled near the base of the POC1B-labeled proximal centriole (95%, 21/22 sperms) ( **Figure 1F, SFig 4, SFig 5, and STable 3** ). However, in cattle, CEP135 ^C^ also labeled to the left of the distal centriole (striated column) (64%, 14/22 sperms). Similarly, CEP135 ^N^ intensely labeled the POC1B-labeled proximal centriole base (95%, 19/20 sperms) and the POC1B-labeled distal centriole left side (striated columns) (85%, 17/20 sperms). Also, CEP135 ^N^ had weak and infrequent labeling at the POC1B-labeled proximal centriole tip (85%, 17/20 sperms) and POC1B-labeled distal centriole right side (striated columns) (55%, 11/20 sperms) ( **Figure 1G, SFig 6, SFig 5, and STable 4** ). Overall, both CEP135 antibodies have common labeling near the cattle spermatozoa centriole base, and the distal centriole left side at the expected location of the striated columns.

Centrosomal coiled-coil protein of 110 kDa (aka CP110 or CCP110) is a centriolar protein with a predicted globular shape (Jumper *et al.* 2021; Varadi *et al.* 2022). CP110 localizes at the distal tips of both mother and daughter centrioles (Figure 6E in Kleylein-Sohn *et al.* 2007) (Yadav *et al.* 2016). CP110 acts as a suppressor of cilia formation by forming a cap over the centriole microtubule tips, restraining the microtubules from growing the cilia/axoneme (Reviewed in Tsang and Dynlacht 2013). As a result, CP110 must be removed from the mother centriole tip for the primary cilium to form (Shen *et al.* 2022).

To label CP110, we used commercial rabbit polyclonal antibodies against CP110’s first 337 amino acids (12780-1-AP). In humans, the CP110 antibody labels the base of POC1B-labeled proximal centriole (73%, 22/30) above the POC1B-labeled proximal centriole (capitulum) (90%, 27/30) and to the left of the POC1B-labeled distal centriole (striated columns) (37%, 11/30, mostly patient 3). ( **Figure 1E, SFig 7, SFig 2, and STable 5** ). Similar labeling was observed in cattle. Intense labeling was observed above the POC1B-labeled proximal centriole (capitulum) (100%, 20/20 sperms) and near the left side of the POC1B-labeled distal centriole (striated column) (100%, 20/20 sperms). Weaker labeling was observed near the right side of the POC1B-labeled distal centriole (striated column) (65%, 13/20 sperms) ( **Figure 1H, SFig 8, SFig 5, and STable 6** ). Overall, CP110 antibodies have common labeling near the human and cattle spermatozoa centrioles at the expected location of the capitulum and striated columns.

CEP135 and CP110 are well-characterized components of canonical centrioles with a conserved evolutionary function. CEP135 is in the base of canonical centrioles, where it promotes centriole microtubule growth (Lin *et al.* 2013; Noga *et al.* 2022), and CP110 is in the tip of the centriole where it inhibits ciliogenesis (Yadav *et al.* 2016). Our results are consistent with CEP135 localization at the proximal centriole base. However, it is surprising that CEP135 in cattle and CP110 in humans and cattle are also found near the centrioles in the striated columns and capitulum. This out-of-the-centriole localization suggests that CEP135 and CP110 may have a novel function in spermatozoa. One possibility is that CP110 and CEP135 have a structural role in the striated columns and capitulum. The sperm centrosome and centriole are modified during spermiogenesis in processes named centrosome reduction (reviewed in Manandhar *et al.* 2000) and centriole remodeling (Khire *et al.* 2016). As part of these processes, the striated columns and capitulum are formed (Fawcett and Phillips 1969) . It is likely that during these processes, centriolar proteins eliminated from the proximal centriole and distal centriole appear in the striated columns and capitulum.

Another interesting result is the difference in CEP135 localization between humans and cattle. One possible explanation for that is the known differences in the size of the sperm distal centriole and striated columns between mammalian species (Khanal *et al.* 2021; Leung *et al.* 2021). These differences may have evolved because sperm cells are subject to intense evolutionary pressure and can vary wildly from species to species (Torgerson *et al.* 2002). Different evolutionary pressures could have forced the specialization of male spermatozoa centrioles (Khanal *et al.* 2023). Finally, it is interesting that CEP135 and CP110 have heterogeneous localization in the striated columns and capitulum, suggesting these structures have specialized domains.

## Methods

**Sperm preparation**

For cattle : Sperm was provided by Select Sires Incorporated as a frozen straw. Straws were stored in liquid nitrogen until use. A 100 mL graduated cylinder was filled with 37°C water, and the straws were plunged in. High-quality sperm was selected using differential centrifugation according to the manufacturer’s recommendations. (PureSperm),

Briefly, all PureSperm gradient solutions as well as Medium 199, were warmed to to 37°C. One mililiter (1.0mL) of 80% PureSperm was added to a 15 mL conical tube as the bottom phase, followed by gentle layering of 1.0 mL of 40% PureSperm, as the upper phase, taking care not to mix the phases. Approximately 0.5 mL of preserved sperm sample was placed on the top of the upper phase. The tube was then centrifuged for 20 minutes at 400 x g. All solutions were pipetted off and discarded, leaving the pellet intact. The pellet was resuspended into 2.0 mL PureSperm wash medium. The resuspended pellet was centrifuged for eight minutes at 250 x g, and the supernatant (approximatly 1.9 mL) was taken from the tube without disrupting the pellet. The pellet was resuspended in 0.1 mL of Medium 199 media.

For humans: Semen samples were obtained from the University of Michigan RSRSR (Schon *et al.* 2021) (Jaiswal *et al.* 2022) from de-identified, fertile men (prior reported history of at least one pregnancy, normal semen analysis, and no reported history of hormonal treatments). The semen samples were produced via masturbation and ejaculated into containers within a private clinic room. The ejaculates were permitted to liquefy for at least 30 min at 37°C, followed by a basic semen analysis following WHO guidelines 5th ed., 2010) This included information on semen volume, sperm count, motility, and morphology (WHO 2010) Samples were separated into seminal and sperm fractions, frozen in sperm cryopreservation media (TYB, Irvine Scientific), according to the manufacturer’s instructions, until needed or were preserved using Sperm CryoProtect (Nidacon, SCP-020) and kept in liquid nitrogen until use. Human sperm samples were acquired with approval from the University of Toledo’s Institutional Review Board (IRB) and the University of Michigan Medical School Institutional Review Boards (IRBMED), the latter registered under IDHUM00125627.

**Slide preparations**

For cattle and humans : About 10 μL of suspended sperm from cattle or 20 μL from human sperm suspension were pipetted onto a slide. A coverslip was placed on the respective sperm droplets and then it was placed in liquid nitrogen. After snap-freezing in liquid nitrogen, the slides were kept in a liquid nitrogen tank until needed.

**Sperm staining**

For cattle and humans: The slides were placed in a pre-chilled Coplin jar with methanol at –20°C for two minutes for cattle or five minutes for humans to fix the sperm to the slides. The slides were then transferred to a PBS wash medium at room temperature, followed by permeabilization with PBST for 60 minutes. Blocking was done using PBSTB for 30 minutes. The primary antibodies diluted in PBSTB were then added and covered with a piece of parafilm, followed by overnight incubation in a humidified chamber at 4°C (see Tables 1-3 for dilutions). The slides were washed in PBST three times for five minutes each. Secondary antibodies and Hoechst in PBSTB were then added to the slide and incubated for two hours at room temperature (See Tables 1-3 for dilutions). The slides were washed three times in PBST for five minutes and three times in PBS for five minutes. A drop of mounting media and a cover slide were added. The slides were sealed with nail polish and stored in a fridge at 4°C until imaging.

**Sperm visualization**

**Confocal** :

For cattle : Slides were visualized using a Leica SP8 confocal microscope in BrightR mode using an HC PL APO CS2 63x/1.40 OIL lens, 100% gain, 1024 × 1024 pixels (62 μM x 62 μM) format, 3x zoom factor, maximum line averaging of 3, frame accumulation of 2, and occasional rotation. Four sequences were used to collect the fluorescence signals. DNA and phase-like images were produced in the first sequence using a 410 nm laser, the emissions were captured using the HyD1 detector to detect photons between 415-478nm maximum and was color-coded to blue, To get a phase-like image, we set the fluoro-turret to Scan-PH, and PMT Trans was set to ON with a gain of 250 in greyscale. CEP135 or CP110 images in the second sequence, the anti-rabbit secondary was conjugated ALEXA 488, we activated a 488 nm laser. We set the absorption spectrum to 493-551nm maximum via the HyD3 or HyD4 detector, and it was color-coded to green. Tubulin images were in the third sequence, the anti-sheep secondary was conjugated to ALEXA 555, we set a 561 nm laser. We set the absorption spectrum to 556-623nm maximum via the HyD3 or HyD4 detector, and it was color-coded to red POC1B in the fourth sequence, the anti-mouse secondary was conjugated to ALEXA 647, we activated a 633 nm laser. The absorption spectrum was set to 638–710nm maximum via the HyD4 detector and was color-coded to magenta. We collected between 10-20 Z-sections of 0.3 μM thickness from the top to the bottom of the sperm.

For humans : Slides were visualized using a Leica SP8 confocal microscope in BrightR mode using an HC PL APO CS2 63x/1.40 OIL lens, 100% gain, 1024 × 1024 pixels (62 μM x 62 μM) format, 3x zoom factor, maximum line averaging of 3, frame accumulation of 2, and occasional rotation. Three sequences were used to collect the fluorescence signals. DNA and phase-like images were produced in the first sequence using a 410 nm laser, the emissions were captured using the HyD1 detector to detect photons between 415-478 maximum and was color-coded to blue, To get a phase-like image, we set the fluoro-turret to Scan-PH, and PMT Trans was set to ON with a gain of 250 in greyscale. CEP135 or CP110 and POC1B images in the second sequence, the anti-rabbit secondary was conjugated ALEXA 488, we activated a 488 nm laser. POC1B in the second sequence was acquired by activating the anti-mouse secondary was conjugated to ALEXA 647 with a 633 nm laser, the absorption spectrum was set to 651–695 nm via the HyD4 detector and was color-coded to magenta. Tubulin images in the third sequence, the anti-sheep secondary was conjugated to ALEXA 555, we set a 561 nm laser and the absorption spectrum to 566-623nm via the HyD3 detector and was color-coded to red. We collected between 10-20 Z-sections of 0.3 μM thickness from the top to the bottom of the sperm.

**HyVolution:**

For cattle : We used similar conditions as above except 6x zoom factor and used standard mode and gain between 10%-40%. Images for Figure 1B -D were deconvoluted using HyVolution II from Leica Microsystems. The SVI Huygens Essential program was used with the typical strategy, no auto-cropping was done, and the mounting media refractive index was set for Vectashield (1.457).

For humans : We used similar conditions as above except 6x zoom factor, used standard mode, set gain between 10%-40%, and two sequences were used to collect the fluorescence signals. DNA and tubulin images were produced in the first sequence, and CEP135 or CP110 and POC1B images in the second sequence. No phase-liked image was collected. Images were deconvoluted using HyVolution II from Leica Microsystems. The SVI Huygens Essential program was used with the typical strategy, no auto-cropping was done, and the mounting media refractive index was set for Vectashield (1.457).

A summary of the HyVolution observations is found in **Stable 7**

**Image preparation and analysis**

**Confocal:**

For cattle and humans : TIF images generated by confocal microscopy were imported into Adobe Photoshop. Photoshop was used to increase intensity and rotate the images of the sperm, with the head rostral, tail caudal, and reflected such that the proximal centriole was oriented to the right as needed. We made a 500 x 1000-pixel low magnification image including the head, neck, and tail; a medium magnification of 150 x 300-pixel image including the head, neck, and centrioles; and a high magnification 75 x 75-pixel image to focus on the centrioles. Images were adjusted to the size of 300 DPI with dimensions of 1 in x 2 in for low and medium magnification and 1 in x 1 in for high magnification. The images were saved as a Photoshop file and then exported to Adobe Illustrator, where they were formatted, arranged, and labeled.

**HyVolution:**

For cattle and humans : TIF images were generated as above at 75 x 75 pixels. Images were adjusted to the size of 300 DPI with dimensions of 0.667 in x 0.667 in.

## Reagents

**Primary Antibodies:**

Anti-POC1B made in Mouse (Thermo Fisher Scientific, H00282809-B01P). This mouse polyclonal antibody was raised against a full-length human POC1B (aka WDR51B). The antibody specificity was demonstrated in (Turner, Katerina A., et al. 2022) . Anti-Tubulin made in Sheep (Cytoskeleton, Inc. ATN02). The specificity of the antibody was demonstrated in the references (Piroli et al. 2014; Lobert et al. 2022). Commercial anti-CEP135 made in Rabbit (Thermo Fisher Scientific, 24428-1-AP). This rabbit polyclonal antibody was raised against the first 233 amino acids of human CEP135. The antibody specificity was demonstrated (Gupta H. et al. 2020). Dilutions are found in **STable 2 & STable 4** . Anti-CEP135 (custom) made in Rabbit (Tang K Tang PMID: 23511974) against amino acids 650-1140. Dilutions are found in **STable 1 & STable 3** . Anti-CP110 made in Rabbit (Thermo Fisher Scientific, 12780-1-AP) This rabbit polyclonal antibody was raised against the first 337 amino acids of CP110 human. Dilutions are found in **STable 5 & STable 6** . Antibodies were validated by staining spindle poles and centrosomes of human Hela cells ( **SFig 9).**

**Secondary Antibodies:**

Anti-Mouse Alexa 488 made in Donkey (Thermo Fisher Scientific, A-21436) (Diluted cattle and human 1:400). Anti-Sheep Alexa 555 made in Donkey (Thermo Fisher Scientific, A-21436) (Diluted cattle and human 1:1000). Anti-Rabbit Dylight 650 made in Donkey (Thermo Fisher Scientific, SA5-10041) (Diluted cattle and human 1:400). Anti-Mouse Alexa 647 made in Donkey (Thermo Fisher Scientific, 715-605-150) (Diluted cattle 1:400). Anti-Rabbit Alexa 488 made in Donkey (Thermo Fisher Scientific, 711-545-152) (Diluted cattle 1:400.)

**Solutions:**

Washing solution: PBS

Permeabilization Buffer (PBST): is made of PBS with 0.3% Triton X-100 (Sigma Aldrich, 9002-93-1)

Blocking Solution (PBSTB): Is made of PBST with 1% BSA (Bovine Serum Albumin) (CHEM-IMPEX INT’L, 00535)

Cattle sperm: Cryopreserved cattle sperm in straws obtained from Select Sires Inc.

Human sperm: Cryopreserved human sperm in vials.

40/80 Density gradient: 40% Nidacon Cat # PS40-100, 80% Nidacon Cat # PS80-100

Sperm washing media: Nidacon, Cat # PSW-100

Sperm resuspension media: M199 (Sigma-Aldrich, Cat # M7528-500ML)

Fixation media: Methanol (-20°C) (Fisher Chemical, A412P-4)

Mounting media: Fluoroshield with DAPI (Sigma-Aldrich, F6057-20ML)

Hoechst Stain: Thermo Fisher Scientific, H1399 (10 mg/mL) (Diluted 1 to 2000)

Slide sealing media: Nail polish, EMS Diasum, 72180

**Materials**

Slides and Cover Slips: Glass slide (Azer Scientific, EMS200A+), glass coverslip (VWR, 48366-205)

Glass Coplin jars: Research Products International Corp. Cat. # 50-212-281
